# Construction of a novel gene signature linked to ferroptosis in pediatric sepsis

**DOI:** 10.3389/fcell.2025.1488904

**Published:** 2025-02-25

**Authors:** Mingyuan Fan, Meiting Chen, Yongqi Gao, Huilin Jiang, Yanling Li, Gongxu Zhu, Shengkuan Chen, Yiming Xu, Xiaohui Chen

**Affiliations:** ^1^ Department of Emergency, The Second Affiliated Hospital, Guangzhou Medical University, Guangzhou, Guangdong, China; ^2^ School of Basic Medical Sciences, Guangzhou Medical University, Guangzhou, China

**Keywords:** pediatric sepsis, ferroptosis-related genes, immune infiltration, ceRNA network, gene expression omnibus data base

## Abstract

**Introduction:**

Pediatric sepsis is a complex and life-threatening condition characterized by organ failure due to an uncontrolled immune response to infection. Recent studies suggest that ferroptosis, a newly identified form of programmed cell death, may play a role in sepsis progression. However, the specific mechanisms of ferroptosis in pediatric sepsis remain unclear.

**Methods:**

In this study, we analyzed microarray datasets from pediatric sepsis and healthy blood samples to identify ferroptosis-associated genes. A protein-protein interaction (PPI) network analysis and histological validation were performed to identify key genes. Additionally, immune infiltration analysis was conducted to explore the correlation between immune cells, immune checkpoint-related genes, and key genes. A competing endogenous RNA (ceRNA) network was constructed to investigate potential regulatory mechanisms involving long non-coding RNAs (lncRNAs), microRNAs (miRNAs), and key ferroptosis-related genes.

**Results:**

We identified 74 genes associated with ferroptosis in pediatric sepsis. Among them, five key genes (MAPK3, MAPK8, PPARG, PTEN, and STAT3) were confirmed through PPI network analysis and histological validation. Immune infiltration analysis revealed significant interactions between immune cells and key genes. The ceRNA network provided insights into the regulatory relationships between lncRNAs, miRNAs, and ferroptosis-related genes.

**Discussion:**

These findings enhance our understanding of the role of ferroptosis in pediatric sepsis and highlight potential therapeutic targets for future research and clinical interventions.

## 1 Introduction

Sepsis, a potentially fatal disease, poses a significant threat to public health. This is mainly due to its link with an abnormal immune response to infections, leading to severe organ dysfunction (Cecconi et al., 2018). Globally, it is estimated that there are 22 cases of pediatric sepsis per 100,000 person-years, along with 2202 cases of neonatal sepsis per 100,000 live births, resulting in a total of 1.2 million cases of pediatric sepsis annually (Fleischmann-Struzek et al., 2018). A considerable number of children suffering from sepsis encounter unresponsive shock and/or multiple organ dysfunction syndrome, leading to a high mortality rate within the first 48–72 h of treatment (Morin et al., 2016; Weiss et al., 2020). To improve outcomes for pediatric patients diagnosed with sepsis, it is essential to quickly identify, administer appropriate resuscitation, and provide comprehensive care.

Ferroptosis is a regulated form of cell death that is specifically influenced by iron-dependent lethal lipid peroxidation. Morphologically, ferroptosis is characterized by the disappearance of mitochondrial cristae and the condensation and rupture of mitochondrial membranes (Hirschhorn and Brent, 2019).

Intracellular iron-dependent lethal lipid peroxidation involves iron overload, ROS generation, and increased levels of polyunsaturated fatty acids in phospholipids. These processes result in cell membrane disruption, impaired membrane function due to lipid cross-linking, and oxidative damage to cellular components, ultimately leading to cell death (Huo et al., 2019; Zhao et al., 2019). In sepsis, bacterial infection relies on iron for their growth within the host organism. The release of intracellular iron during infection serves as a rich resource for bacterial utilization (Kleist and Knoop, 2020). This process, combined with the generation of reactive oxygen species and fatty acids by bacterial infection, fuels lipid peroxidation (Wood et al., 2018). This creates a detrimental cycle that worsens the infection and ultimately leads to sepsis, which can result in multi-organ dysfunction (Burke et al., 2019). In summary, ferroptosis plays a significant role in exacerbating infections by providing multiple sources for bacterial growth, making it a critical cascade in sepsis.

This study sourced gene expression datasets from the Gene Expression Omnibus (GEO). Bioinformatics analyses were employed to identify differentially expressed genes (DEGs). By integrating these DEGs with ferroptosis-related genes (FRGs), we pinpointed five key genes: MAPK3, MAPK8, PPARG, PTEN, and STAT3. To underscore the importance of these hub genes, we used the other independent dataset for validation.

## 2 Materials and methods

### 2.1 Data collection and acquisition of genes related to ferroptosis

Microarray data were sourced from the Gene Expression Omnibus (GEO) database, specifically from two series: GSE145227, and GSE13904. The GSE145227 series, which was used as the training set, contains 22 total RNA samples derived from whole blood, including 10 samples from children with sepsis and 12 samples from healthy controls. The GSE13904 series, used as the validation set, includes 52 whole blood samples from children with sepsis and 18 samples from healthy controls. For subsequent analyses, we gathered a total of 564 experimentally validated FRGs involved in the regulation, inhibition, or identification of ferroptosis. These were obtained from the publicly accessible FerrDb database (http://www.zhounan.org/ferrdb/), after removing any duplicated genes.

### 2.2 Discovering differential expression of ferroptosis-associated genes

We re-annotated the series matrix using the “AnnoProbe” package. We then normalized the microarray data and identified differentially expressed genes (DEGs) by comparing sepsis samples and healthy control, using the “limma” package in R software. We considered DEGs with an absolute value of |log2FC| ≥ 1 and an adjusted p-value <0.05 as significant. Furthermore, we applied the same criteria to identify differentially expressed long non-coding RNAs (DElncRNAs). To identify the genes that overlapped between the DEGs and FRGs, we defined them as differentially expressed ferroptosis-related genes (DE-FRGs). Subsequently, we generated volcano plots to visualize the expression patterns of both DEGs and DElncRNAs, using the “ggplot2” package. We also used the “Venndiagram” package to represent the number of DE-FRGs in a Venn diagram. Finally, we demonstrated the expression levels of DE-FRGs through a heatmap, again utilizing the “ggplot2” package.

### 2.3 Gene set enrichment analysis in child sepsis

To uncover the relevant signaling pathways implicated in the progression of sepsis, gene set enrichment analysis (GSEA) was conducted using the online tool OmicStudio (http://www.omicstudio.cn/tool). Gene sets that exhibited significant enrichment, meeting the criterion of a nominal (NOM) p-value <0.05 and false discovery rate (FDR) <25%, were deemed statistically significant and were subsequently highlighted.

### 2.4 Functional and pathway enrichment of DEGs and DE-FGRs

We utilized the “clusterProfiler” package in R to perform enrichment analyses on DEGs and DE-FRGs. Our analysis, using Gene Ontology (GO), was designed to reveal biological functions in three distinct categories: biological process (BP), cellular component (CC), and molecular function (MF). Additionally, we carried out an analysis using the Kyoto Encyclopedia of Genes and Genomes (KEGG) to explore potential pathways.

### 2.5 Construction of protein–protein interaction network of DE-FRGs

Using the STRING online website (https://string-db.org/), we conducted a protein-protein interaction (PPI) network analysis of the differentially expressed genes (DEGs) related to ferroptosis. The obtained results were imported into Cytoscape (version 3.8.2), and relevant subnetworks were extracted using the MCODE plugin. The top five genes in the PPI network were identified as hub genes based on their rankings using the maximum neighborhood component (MNC), degree, and edge percolated component (EPC) algorithms. These calculations were performed with the cytoHubba plugin.

### 2.6 Immune infiltration and immune checkpoint genes between sepsis and controls

The progression of sepsis is closely linked to the body’s immune response to harmful microorganisms. To investigate the differential expression of markers on immune cells between the sepsis and control groups, we utilized the CIBERSORT tool. This tool applies the principles of linear support vector regression to decode the matrix of expressed genes related to various human immune cell subtypes. The analysis was conducted using a reference dataset comprising gene expression features for 22 unique subtypes of immune cells. Furthermore, we investigated the differences in the expression of immune checkpoint genes between the sepsis and control groups.

### 2.7 Validation of hub gene expression in sepsis datasets

The microarray dataset of sepsis (GSE13904, n = 70, normal vs. sepsis) was downloaded from the GEO database to verify the expression of the hub genes. The “limma” package was used to identify the DEGs with thresholds of *|*log2FC*|* ≥ 1 and adjusted p < 0.05.

### 2.8 Sepsis animal model procedure

The animal procedure was reviewed and approved by the Animal Ethics Committee of the Second Affiliated Hospital of Guangzhou Medical University (permit no. B2024-063). Eight-week-old C57BL6 mice (purchase from Guangzhou YanCheng Biotechnology Co., Ltd., Guangzhou, China) were then randomly assigned to two groups. Sepsis was induced in the mice using the cecal ligation and puncture (CLP) method, following established protocols (Wang et al., 2022). Mice were sedated with isoflurane, and a 1 cm midline abdominal incision was made. The cecum was exteriorized, and half of it was ligated using 4–0 silk. A 21-gauge needle was used to puncture the cecum, and patency was confirmed by gentle pressure. The cecum was then returned to the abdominal cavity, and the incision was closed in layers. All mice received a subcutaneous injection of isotonic sodium chloride solution (5 mL/100 g) to prevent dehydration. Sham-operated mice underwent the same procedure without the puncture. All mice were euthanized 24 h post-operation, and tissue samples from the heart were collected.

### 2.9 Hematoxylin and eosin (H&E) staining

Murine tissues were collected and fixed in 4% paraformaldehyde for 24 h prior to OCT embedding. The embedded blocks were then sectioned into 5 μm cryosections and fixed in 4% PFA for an additional 10 min. The prepared slides underwent H&E staining. Whole-slide images were captured using an Aperio CS2 Digital Pathology Scanner (Leica Biosystems, USA) and converted to TIFF format. The histological scoring was defined as follows: 0, no myocardial lesions; +1, lesions involving up to 25% of the myocardium; +2, lesions involving 25%–50% of the myocardium; +3, lesions involving 50%–75% of the myocardium; and +4, lesions involving more than 75% of the myocardium (Kishimoto scores) (Kishimoto et al., 2001). Five sections from each group were analyzed.

### 2.10 Real-time quantitative PCR

Total RNA was extracted using AG RNAex Pro Reagent (Cat No: AG21102; Accurate Biology). The RNA concentration was measured using a Nanodrop 2000 spectrophotometer. Subsequently, RNA was reverse transcribed into cDNA using the Evo M-MLV Mix Kit (Cat No: AG11728; Accurate Biology), following the manufacturer’s instructions. qPCR was performed on a CFX-96 Real-time System (BioRad) instrument, utilizing the SYBR Green Premix Pro Taq HS qPCR Kit (Cat No: AG11701; Accurate Biology). The relative expression of the target genes was calculated using the 2^(-ΔΔCt) method, with Gapdh as the reference control. The specific primer sequences used, synthesized and desalted by Generay Biotechnology, are shown in Table 1.

**TABLE 1:** Primer sequences.

Gene name	Sense	Antisense
Mapk3	TCC​GCC​ATG​AGA​ATG​TTA​TAG​GC	GGT​GGT​GTT​GAT​AAG​CAG​ATT​GG
Mapk8	AGC​AGA​AGC​AAA​CGT​GAC​AAC	GCT​GCA​CAC​ACT​ATT​CCT​TGA​G
Pparg	TCG​CTG​ATG​CAC​TGC​CTA​TG	GAG​AGG​TCC​ACA​GAG​CTG​ATT
Pten	TGG​ATT​CGA​CTT​AGA​CTT​GAC​CT	GCG​GTG​TCA​TAA​TGT​CTC​TCA​G
Stat3	CAA​TAC​CAT​TGA​CCT​GCC​GAT	GAG​CGA​CTC​AAA​CTG​CCC​T
Gapdh	AGG​TCG​GTG​TGA​ACG​GAT​TTG	TGT​AGA​CCA​TGT​AGT​TGA​GGT​CA

### 2.11 Western blot

Proteins were isolated from the tissue samples following a standard protein extraction protocol. The extracted proteins were separated by sodium dodecyl sulfate-polyacrylamide gel electrophoresis (SDS-PAGE) based on their molecular weight. After separation, the proteins were transferred to a polyvinylidene fluoride (PVDF) membrane. The membrane was blocked for 1 h at room temperature with Tris-buffered saline and Tween-20 (TBST) containing 5% nonfat milk powder. The membrane was incubated with primary antibodies targeting the proteins of interest. The primary antibodies used were as follows: Gpx4 (1:10,000, Cat No: ET1706-45), Mapk3 (1:1000, Cat No: ET1604-32, Huabio), Mapk8 (1:1000, Cat No: R1309-1, Huabio), Pparg (1:1000, Cat No: GB112205-100, Servicebio), Pten (1:1000; Cat No: ET1606-43, Huabio), and Stat3 (1:1000; Cat No: ET1607-38, Huabio). Immunoreactivity was detected using enhanced chemiluminescence (ECL), which visualizes specific proteins through the binding of horseradish peroxidase (HRP) to its substrate. Western blot images were captured using an automated digital gel imaging system.

### 2.12 Construction of the lncRNA–miRNA–mRNA ceRNA network and TFs-hub genes network

Pearson’s correlation was used to conduct co-expression analysis between differentially expressed long non-coding RNAs (DElncRNAs) and hub genes. Only DElncRNA-hub gene pairs with a correlation coefficient above 0.5 and p-value below 0.05 were chosen for further analysis. To reveal potential interactions between lncRNAs and microRNAs (miRNAs), the ENCORI dataset available at starbase.sysu.edu.cn was utilized. Additionally, the ENCORI databases were employed to identify miRNA-mRNA pairs. Consequently, networks comprising lncRNA-miRNA-mRNA interactions were constructed, utilizing five hub FRGs as the basis.

To predict key genes’ transcription factors, we submitted the hub genes to ChIP-X Enrichment Analysis 3 (ChEA3) platform (Keenan et al., 2019). Key genes-associated TFs ranked by mean rank score. Finally, we selected the TFs which score ≤50 as key predicted TFs.

### 2.13 Statistical analysis

Statistical analyses were performed using the R programming language (version 4.1.0) and GraphPad Prism 9.0. Differences between two groups were evaluated using either the Wilcoxon test or Student’s t-test. All statistical P values were two-sided, with significance defined as P < 0.05.

## 3 Result

### 3.1 Identification of DE-FRGs and DE-lncRNAs in child sepsis

**FIGURE 1:**
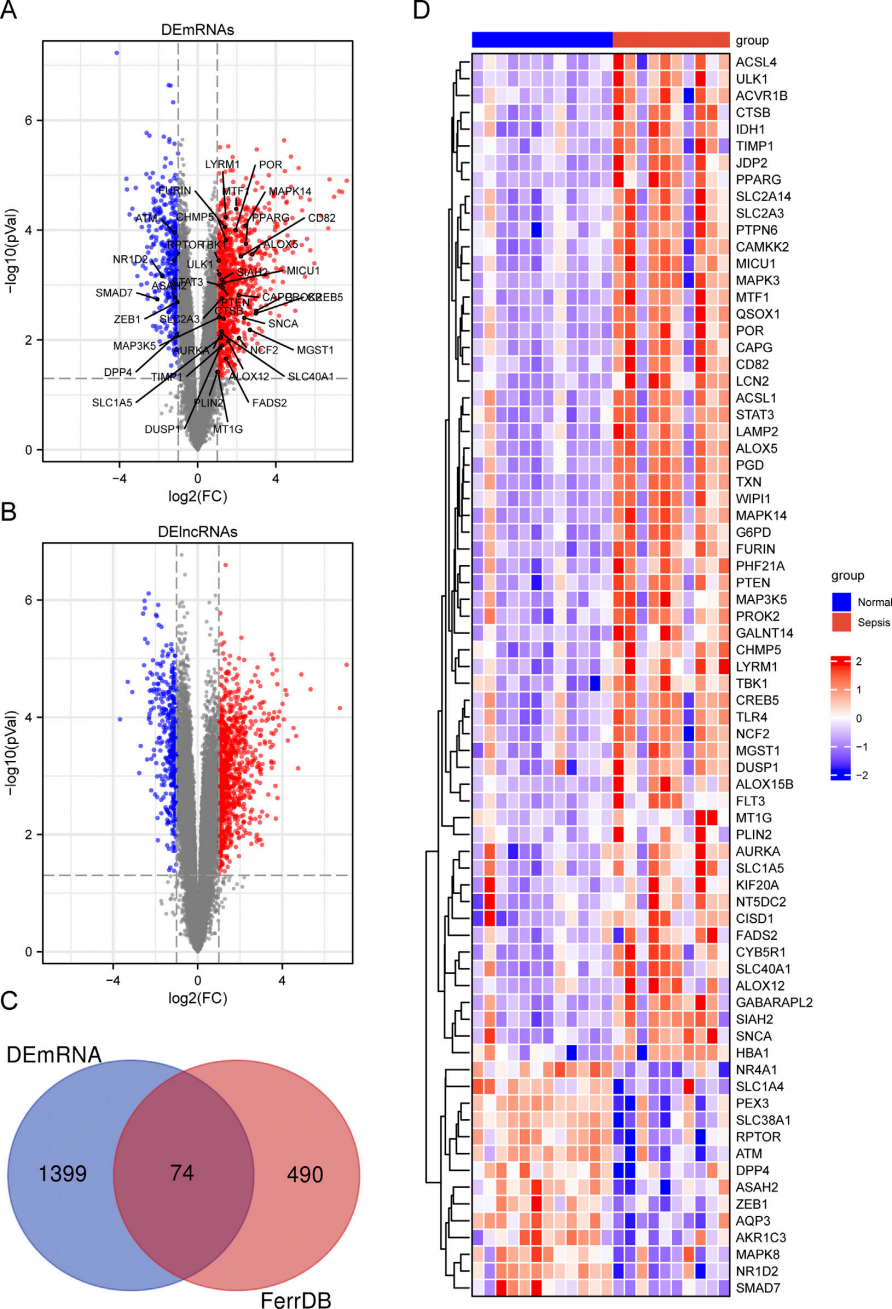
Summary of differentially expressed ferroptosis-related genes (DE-FRGs) and differentially expressed long non-coding RNAs (DElncRNAs). **(A, B)** Volcano plot displaying DEmRNAs **(A)** and DElncRNAs **(B)** in child sepsis. Upregulated genes were denoted by red dots, while downregulated genes were represented by blue dots. The significance thresholds were set at |log2FC| ≥ 1 and adjusted *P* < 0.05. The Venn diagram **(C)** illustrated the overlapping DE-FRGs, and a heatmap **(D)** was generated to display the expressions of the 74 DE-FRGs in child. In the heatmap, the more highly expressed FRGs were indicated by red bricks, while the lower expression was represented by blue bricks.

To investigate the differentially expressed genes linked to ferroptosis in pediatric sepsis, we gathered 564 ferroptosis-related genes (FRGs) from FerrDb, a comprehensive database providing information on ferroptosis regulators, markers, and associated diseases. We then conducted a differential expression analysis on the GSE145227 dataset, revealing 1336 lncRNAs and 1473 genes with significant expression differences between pediatric sepsis samples and healthy blood samples. Our analysis criteria included a minimum absolute log2 fold change of one and an adjusted p-value of less than 0.05, as illustrated in Figures 1A, B. By comparing the DEGs with the FRGs, we identified 74 DE-FRGs that showed differential expression in pediatric sepsis (Figure 1C). To further visualize the expression patterns of these 74 DE-FRGs, we generated a heatmap (Figure 1D; Supplementary Tables S1, S2).

### 3.2 Gene set enrichment analysis

**FIGURE 2:**
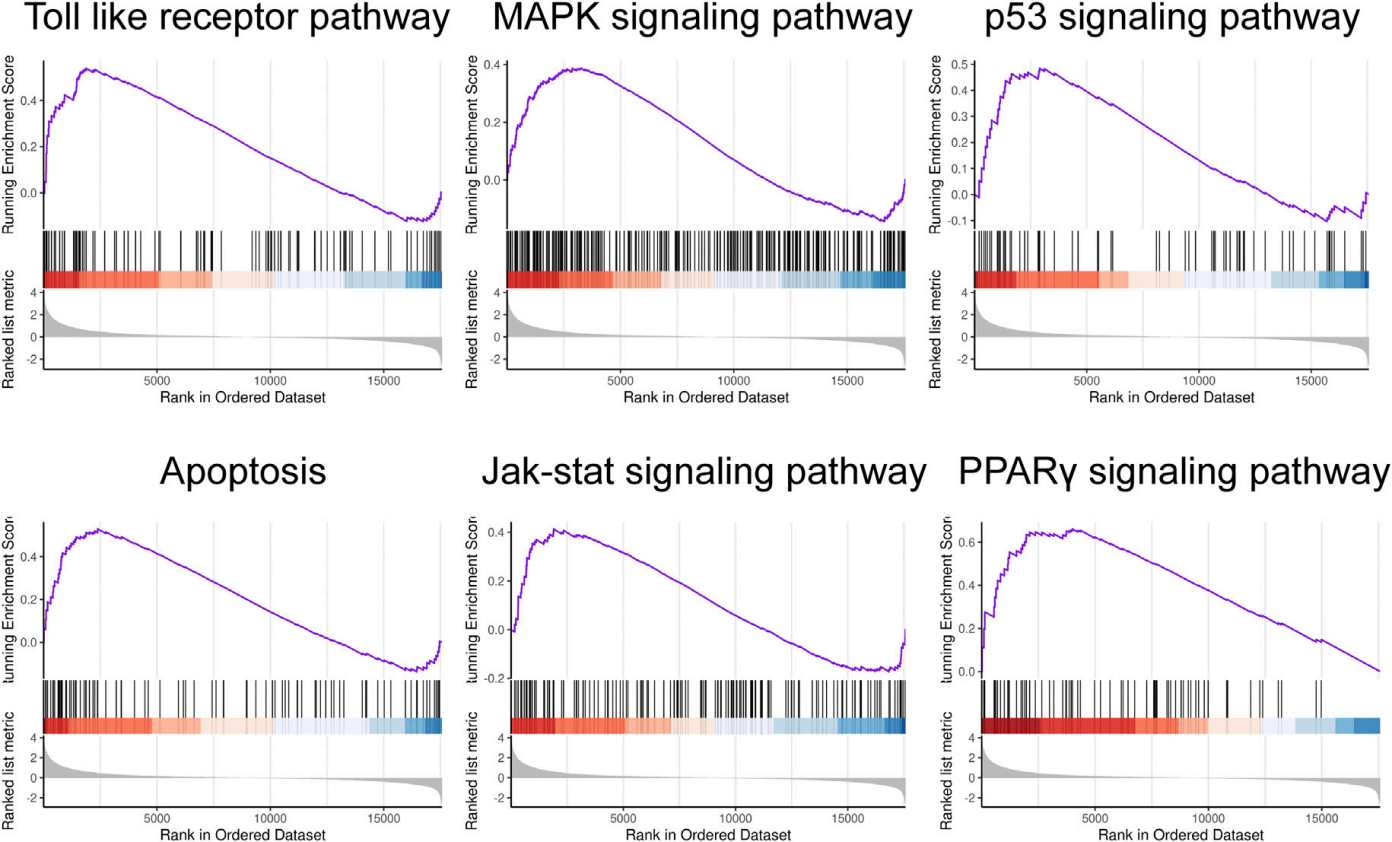
Gene set enrichment analysis. GSEA in child sepsis. NOM *P* < 0.05, FDR <25%.

To assess the distinct pathways utilized by the two groups, we conducted a Gene Set Enrichment Analysis (GSEA) based on 1473 DEGs. The GSEA results revealed significant enrichment of several pathways in pediatric sepsis. These include the Toll-like receptor pathway, the Mitogen-activated protein kinase (MAPK) signaling pathway, the p53 signaling pathway, apoptosis, the Janus kinase (JAK)/signal transducer and activator of transcription (STAT) signaling pathway, and the proliferator-activated receptor γ (PPARγ) signaling pathway (Figure 2).

### 3.3 Functional enrichment analysis of DE-FRGs

**FIGURE 3:**
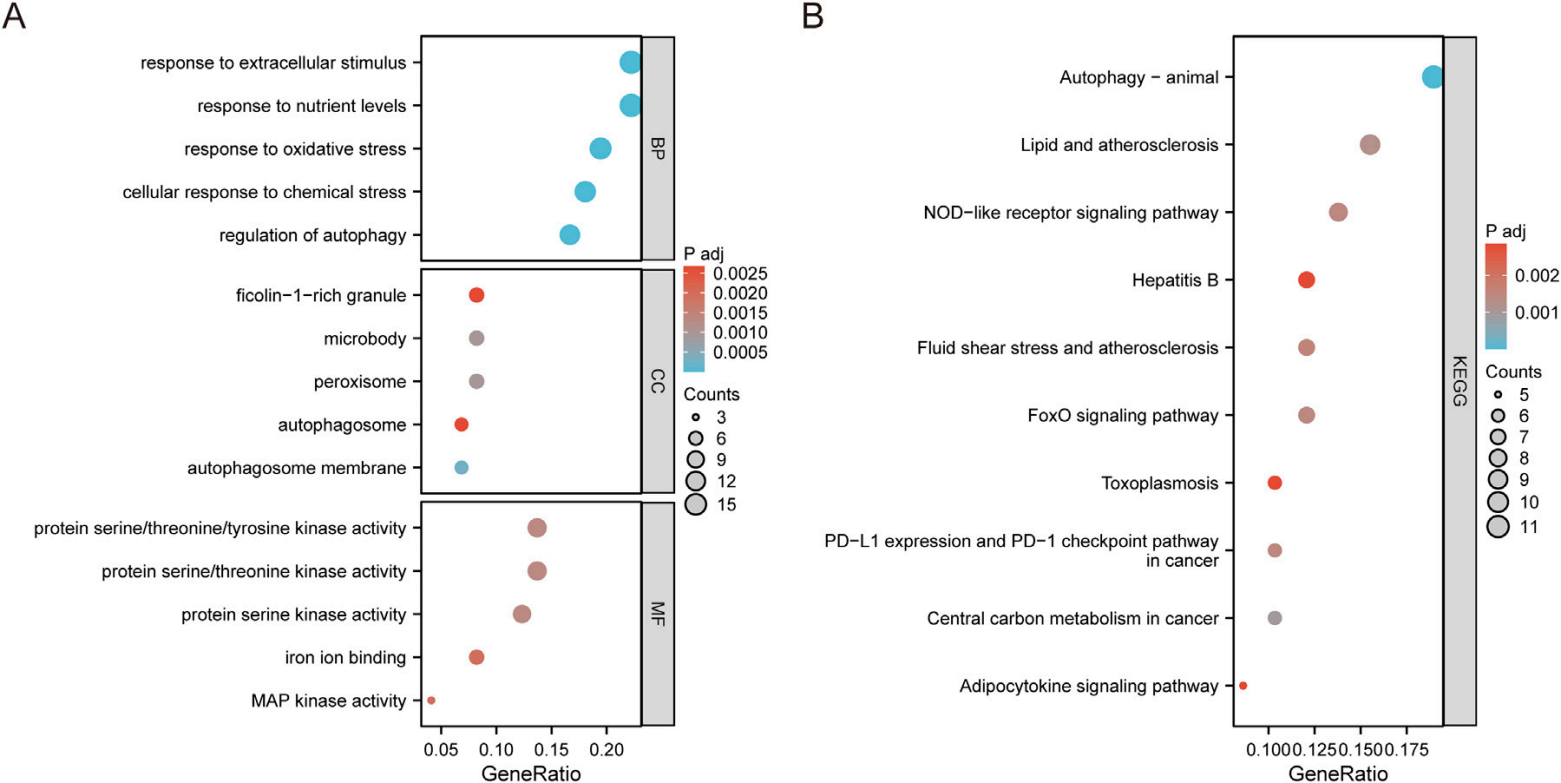
Enrichment analysis of DE-FRGs. **(A)** Top 5 GO (gene ontology) biological processes, cellular component, molecular function pathway. **(B)** Top 10 KEGG pathway.

We utilized GO and KEGG pathway analyses to explore the potential biological functions and pathways linked to the DE-FRGs in both groups. The top 20 results were presented using scatter plots to illustrate enrichment. The GO analysis of DEGs revealed significant enrichment in processes such as positive regulation of cytokine production and immune response-regulating signaling pathway (Supplementary Figure S1A). The KEGG pathway analysis further indicated their involvement in pathways like natural killer cell mediated cytotoxicity, immune cell differentiation, and T cell receptor signaling pathway (Supplementary Figure S1B). A specialized GO analysis was conducted specifically on the DE-FRGs, revealing their involvement with response to oxidative stress, autophagy, iron ion binding, and MAP kinase activity (Figure 3A). As expected, the DE-FRGs exhibited significant enrichment in animal autophagy, NOD-like receptor signaling pathway, and FoxO signaling pathway, as evidenced by the KEGG pathway analysis results (Figure 3B).

### 3.4 Protein–protein interaction network construction and visualization

**FIGURE 4:**
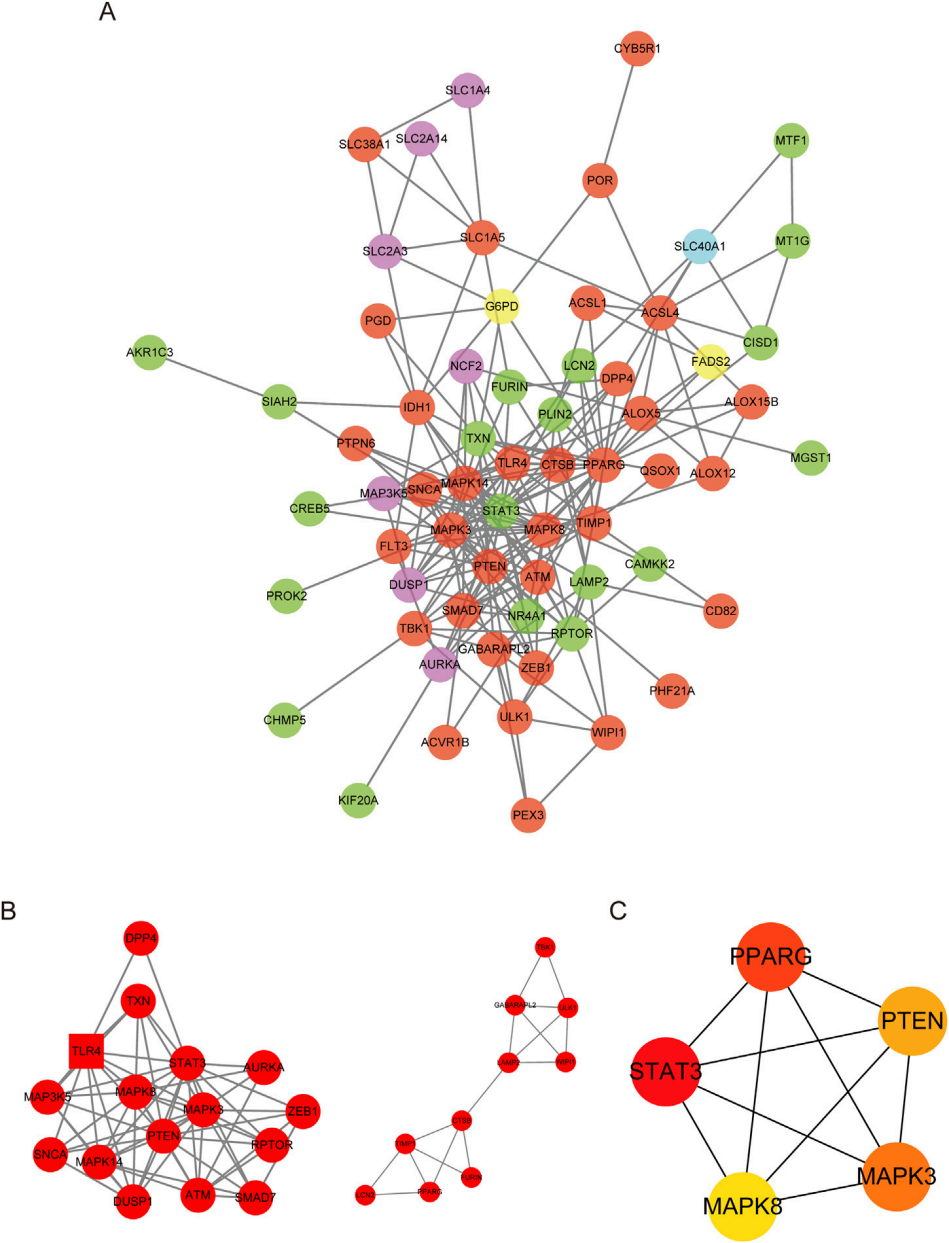
PPI network of DE-FRGs. **(A)** Protein–protein interaction (PPI) network of 74 differentially expressed FRGs (DE-FRGs).Red indicates the driver of ferroptosis, light blue indicates the marker of ferroptosis, and green indicates the suppressor of ferroptosis. **(B)** Two clusters of the PPI network. **(C)** Hub genes calculated using the maximum neighborhood component (MNC), degree, and edge percolated component (EPC) algorithms.

To investigate the interactions among DE-FRGs, we utilized the STING database, a widely recognized resource for protein-protein interactions (PPI). We then constructed and visualized the resulting PPI network using Cytoscape 3.10.0. We then eliminated isolated DE-FRGs, yielding a PPI network with 55 nodes and 223 edges (Figure 4A). With the aid of the MCODE algorithm, the PPI network was divided into two distinct clusters (Figure 4B). The first cluster included sixteen genes (DPP4, TXN, TLR4, MAPK3K5, MAPK8, STAT3, AURKA, SNCA, MAPK14, PTEN, MAPK3, ZEB1, DUSP1, ATM, RPTOR, and SMAD7), while the second cluster contained ten genes (TBK1, GABARAPL2, ULK1, LAMP2, WIPI1, CTSB, TIMP1, FURIN, LCN2, and PPARG). Next, we determined the central genes within the network by selecting the top five genes as determined by the Maximum Neighborhood Component (MNC), degree, and Edge Percolated Component (EPC) algorithms. The selected hub genes were MAPK3, MAPK8, PPARG, PTEN, and STAT3 (Figure 4C).

### 3.5 Examining the association between hub genes and mitochondrial function

Given the association between ferroptosis and mitochondrial activity impairment, we conducted an extensive investigation to establish a link between the hub genes and the genes related to mitochondrial function (MFRGs). Out of the 1899 MFRGs examined, 142 genes were identified as differentially expressed MFRGs (DE-MFRGs) (Supplementary Figure S2A). Among these genes, 114 DE-MFRGs displayed increased expression, while 28 showed decreased expression. Pearson’s correlation analysis demonstrated that a significant number of DE-MFRGs had either positive or negative correlations with the hub FRGs (|r| ≥ 0.5, p < 0.05) (Supplementary Figure S2B).

### 3.6 Differences in immune infiltration and immune checkpoint genes

**FIGURE 5:**
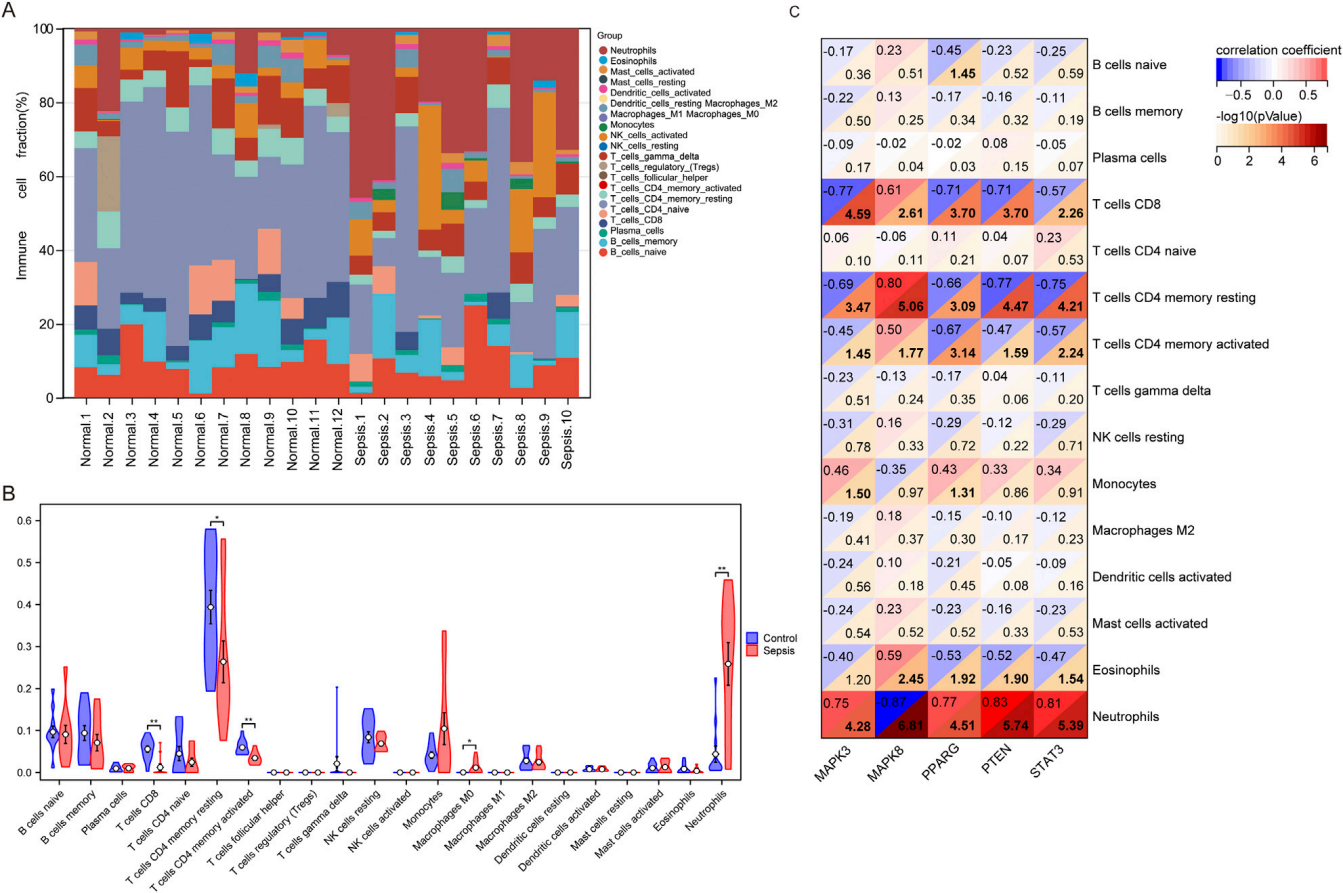
Analysis of immune infiltration in children with sepsis compared to the control group in the GSE145227 dataset. **(A)** Bar graphs illustrating the proportions of 22 immune cell types in individuals with sepsis and the control group. The first 12 bars represent individuals with the control group, while the remaining 10 bars represent the sepsis. **(B)** Differential expression of different types of immune cell marker expression between sepsis and controls. **(C)** Correlation between hub genes and immune cells.

In our comparative study of sepsis and controls, we utilized the CIBERSORT algorithm to conduct a comprehensive analysis of immune cells in peripheral blood for identifying disparities between the sepsis and control cohorts. The expression levels of 22 immune cell markers were evaluated in all patients, with the first 12 patients representing the control group and the remaining 10 patients representing the sepsis group (Figure 5A). Significant variances in the expressions of five immune cell markers were illustrated in Figure 5B. Specifically, the sepsis group demonstrated significantly elevated expression levels of two types of immune cells: M0 macrophages and neutrophils. In contrast, the sepsis group displayed lower expression levels of three immune cell markers: CD8 T cells, resting memory CD4 T cells, and activated memory CD4 T cells.

**FIGURE 6:**
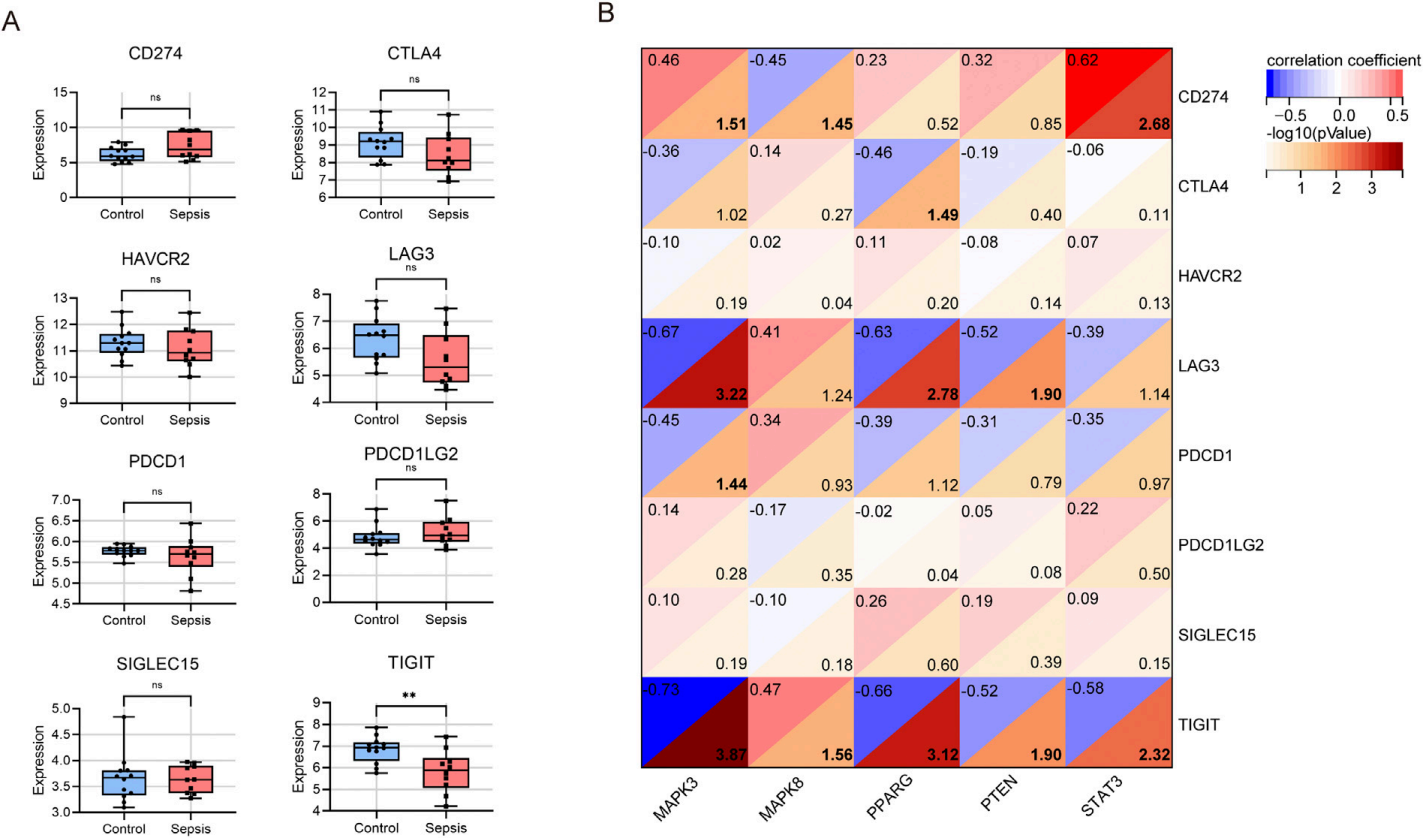
Analysis of immune checkpoint genes in children with sepsis compared to the control group in the GSE145227 dataset. **(A)** Differential expression of different immune checkpoint genes between sepsis and controls. ns no significance; **P < 0.01. **(B)** Correlation between hub gene and immune checkpoint genes.

Next, we utilized microarray data to examine the variation in immune checkpoint gene expression between the sepsis and control groups. We observed downregulation in five genes, namely, CTLA4, HAVCR2, LAG3, PDCD1, and TIGIT. However, only the expression of TIGIT (which codes for T cell immunoreceptor with Ig and ITIM domains) was statistically significant (Figure 6A). Meanwhile, CD274, PDCD1LG2, and SLGLEC15 were upregulated in sepsis, but these changes were not statistically significant.

### 3.7 Correlation of hub genes with immune cells and immune checkpoint genes

To investigate whether hub genes are involved in the immune cell infiltration observed in pediatric sepsis, we carried out a correlation analysis between these hub genes and immune cells. Our findings revealed a negative correlation between the expression level of MAPK8 and the neutrophil count (p < 0.001). Conversely, the expression levels of MAPK3, PPARG, PTEN, and STAT showed a positive correlation with the neutrophil count (p < 0.001; Figure 5C). MAPK3 had significant correlations with five types of immune cells, with the strongest correlation observed in CD8^+^ T cells (r = −0.77, P < 0.001). MAPK8 also correlated significantly with five immune cells, with the CD4^+^ memory resting T cells showing the strongest correlation (r = 0.80, P < 0.001). PPARG showed significant correlations with four immune cells, with CD8^+^ T cells exhibiting the strongest correlation (r = −0.71, P < 0.001). PTEN exhibited significant positive correlations with neutrophil and negative correlations with four others, with the strongest correlation found in neutrophil (r = 0.83, P < 0.001). Similarly, STAT3 displayed significant correlations with five immune cells, with the strongest correlation observed in neutrophil (r = 0.81, P < 0.001). Notably, all the central genes, except for STAT3, were driver genes of ferroptosis, while STAT3 belonged to the suppressor genes of ferroptosis (Figure 5C; Supplementary Tables S2, S4).

In a subsequent analysis of the differential expression of immune checkpoint genes between the sepsis and control groups using microarray data (Figure 6B), we found that MAPK3 had a significant negative association with LAG3 (r = −0.67, P < 0.001), PDCD1 (r = −0.45, p = 0.036), and TIGIT (r = −0.73, p < 0.001), but a positive association with CD274 (r = 0.46, p = 0.031). Conversely, MAPK8 showed a significant negative correlation with CD274 (r = −0.45, P = 0.035) and a positive association with TIGIT (r = 0.47, P = 0.028). PPARG had a negative correlation with CTLA4 (r = −0.46, P = 0.032), LAG3 (r = −0.63, P = 0.002), and TIGIT (r = −0.66, P < 0.001). PTEN showed a negative association with LAG3 (r = −0.52, P = 0.013) and TIGIT (r = −0.52, P = 0.013). Lastly, STAT3 exhibited a positive association with CD274 (r = 0.62, P = 0.002) and a negative association with TIGIT (r = −0.58, P = 0.005). In summary, TIGIT displayed significant negative correlations with four central genes, excluding MAPK8. Additionally, both MAPK3 and STAT3 showed positive correlations with CD274.

### 3.8 The diagnostic potential of hub genes in identifying sepsis in both the training set and validation set

**FIGURE 7:**
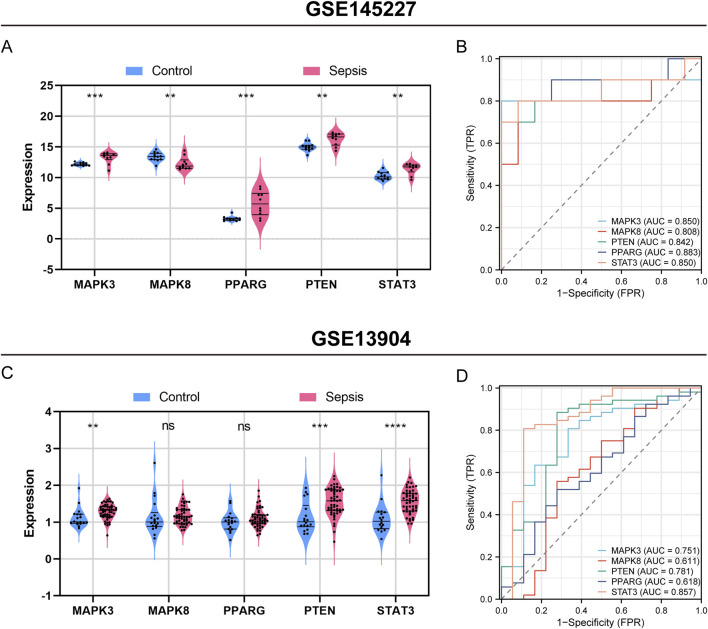
Assessment of hub genes’ diagnostic capability for sepsis in the GSE145227 training set and the GSE13904 validation set. Violin plot displaying expression difference of five hub genes in sepsis and control groups in GSE145227 **(A)**, and GSE13904 **(C)**. Receiver operating characteristic (ROC) curve of five hub genes diagnosis of sepsis in GSE145227 **(B)** and GSE13904 **(D)**. ns, no significance; **, *P* < 0.01; ***, *P* < 0.001, ****, *P* < 0.0001.

Figure 7 illustrates the importance of five hub genes associated with ferroptosis in identifying sepsis within the training set (GSE145227). All five hub genes showed a significant increase in expression levels during sepsis compared to the control group, with MAPK3 and PPARG showing a particularly significant increase (P < 0.001), and the other three genes showing a less significant but still notable increase (P < 0.01) (Figure 7A). The area under the receiver operating characteristic curve (AUROC) for diagnosing sepsis using MAPK3 was found to be 0.850 (95% CI 0.639–1.000), demonstrating a sensitivity of 0.800 and a specificity of 1.000 (Figure 6B, Supplementary Table S5). The AUROCs for diagnosing sepsis using MAPK8, PTEN, PPARG, and STAT3 were calculated as 0.808 (95% CI 0.586–1.000), 0.842 (95% CI 0.643–1.000), 0.883 (95% CI 0.713–1.000), and 0.85 (95% CI 0.652–1.000), respectively. These genes demonstrated sensitivities of 0.900, 0.700, 0.800, and 0.800, and specificities of 0.917, 1.000, 0.917, and 0.917, respectively. Moreover, the positive predictive value (PPV), negative predictive value (NPV), and negative likelihood ratio (NLR) were calculated as 0.000, 0.091, 0.000, and 0.091, respectively. The corresponding positive likelihood ratios (PLR) were calculated as 0.250, 9.000, 0.429, and 0.250 (Figure 7B; Supplementary Table S5).

In a validation set (GSE13904) which includes 52 children with sepsis and 18 healthy individuals, the five hub genes also demonstrated excellent diagnostic performance for sepsis (Figures 7C, D). A violin plot was used to display the expression of the five hub genes in the sepsis or infection and healthy group (Figure 7C). MAPK3, PTEN, and STAT3 were significantly upregulated in pediatric sepsis, consistent with the results of GSE145227. The AUROCs of MAPK3, MAPK8, PPARG, PTEN in the diagnosis of sepsis were 0.751, 0.611, 0.781, 0.618, and 0.857, respectively (Figure 7D).

### 3.9 Validation of the expression of hub genes in animal models

**FIGURE 8:**
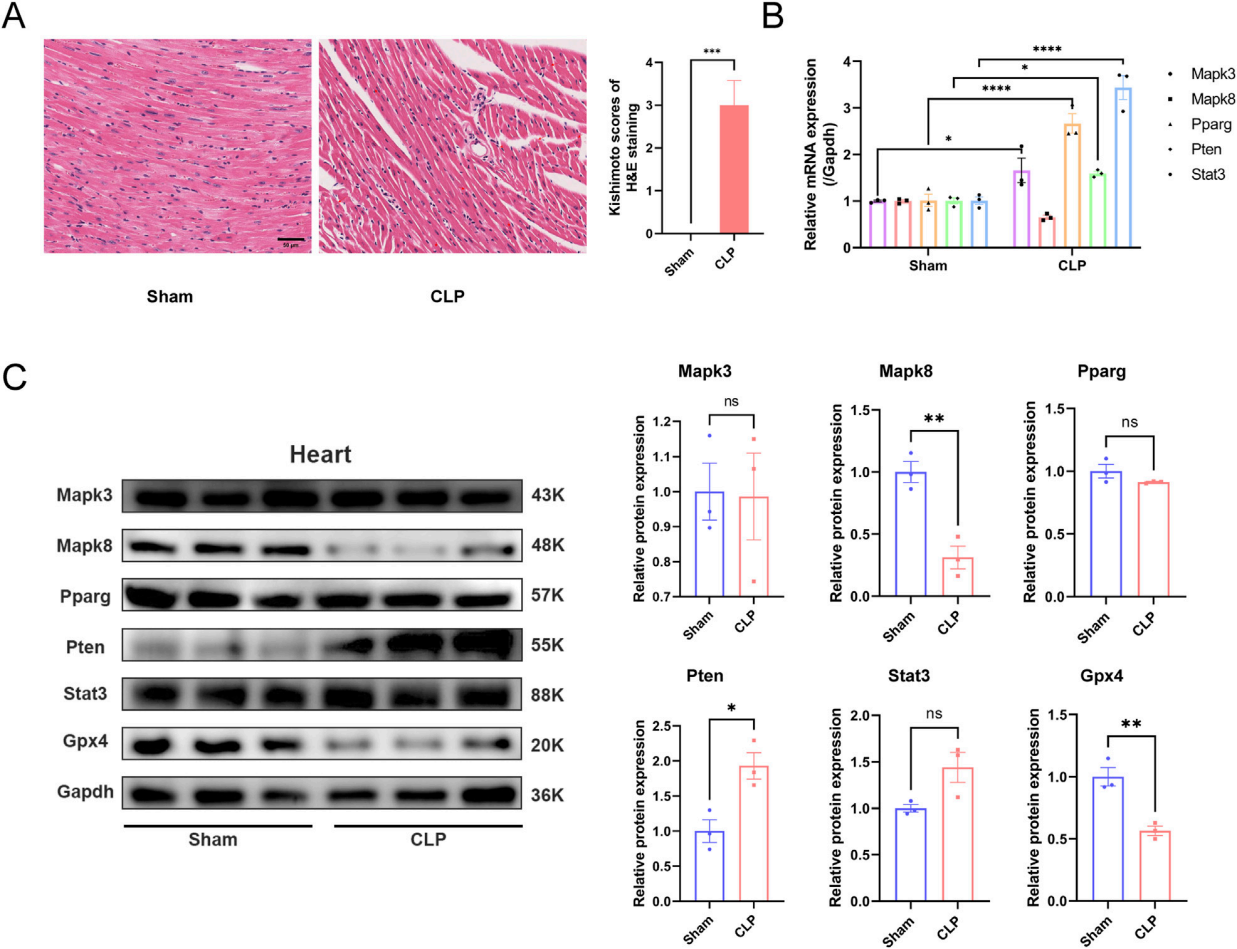
Experimental validation of hub genes in heart tissue in sepsis mice. **(A)** Hematoxylin and eosin (HE) staining reveals the macroscopic morphology of myocardial tissue in mice after CLP surgery. (Kishimoto scores, n = 6 per group, scale bar = 50 μm) **(B)** Expression levels of the five hub genes *in vivo* mice model. **(C)** Protein level of hub genes in sham and CLP group. ns, no significance; *, *P* < 0.05; **, *P* < 0.01; ****, *P* < 0.0001.

The aforementioned bioinformatics analysis identified core genes potentially implicated in sepsis-associated ferroptosis. To substantiate these findings, we conducted validation experiments using a CLP mouse model. In this model, HE staining of cardiac tissue revealed myocardial rupture and separation, accompanied by inflammatory cell infiltration in the CLP group (Figure 8A). Furthermore, we extracted RNA and protein samples from myocardial tissue (Figures 8B, C) to evaluate the expression levels of the identified core genes. Our results demonstrated a significant reduction in GPX4 protein expression in the cardiac tissue of the CLP group, suggesting the occurrence of ferroptosis in the myocardium of septic mice. At the mRNA level, we observed elevated expression of Mapk3, Pparg, Pten, and Stat in the CLP group compared to the Sham group, with the exception of Mapk8, which was downregulated but not statistically significant. At the protein level, the expression of MAPK8 was significantly reduced, whereas the level of PTEN was elevated in the CLP group compared to the Sham group. These findings indicate that these core genes may indeed play a regulatory role in ferroptosis within the cardiac tissue of septic mice.

### 3.10 The lncRNA–miRNA–mRNA ceRNA network and TFs-hub genes network construction

**FIGURE 9:**
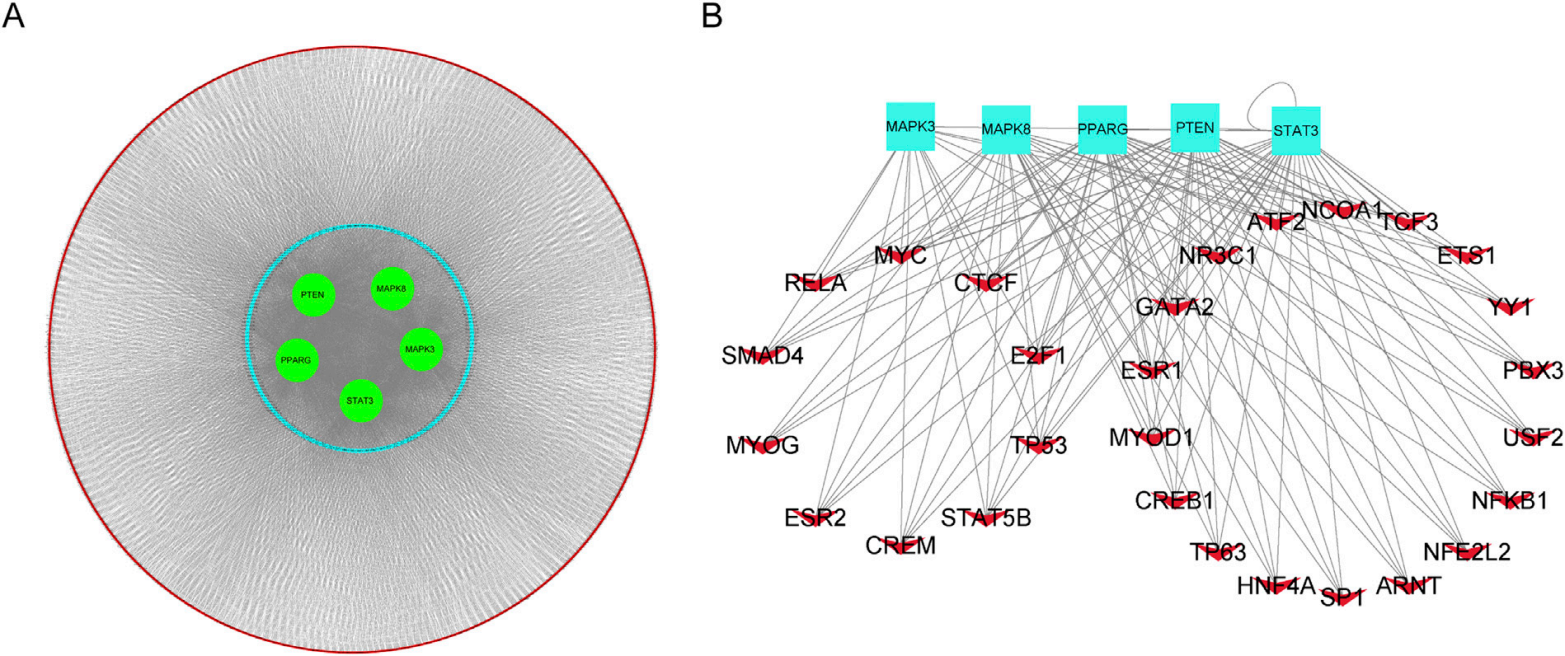
Construction of the competing endogenous RNA (ceRNA) network and TFs-hub genes regulation network. **(A)**. Predicted long non-coding RNAs (lncRNAs) were represented by red nodes, predicted microRNAs (miRNAs) were represented by blue nodes, and hub genes were represented by green nodes. **(B)**. TF-hub genes regulation network, red V nodes represented TFs and hub genes were represented bright blue rectangles.

Leveraging the competitive endogenous RNA (ceRNA) hypothesis, we constructed networks to investigate the role of long non-coding RNAs (lncRNAs) as miRNA sponges in sepsis (Figure 9A). We integrated upregulated lncRNA and hub ferroptosis-related gene (FRG) pairs with co-expression into the upregulated ceRNA network, along with the inclusion of predicted miRNAs. In total, this ceRNA network consists of 3960 lncRNA nodes, 506 miRNA nodes, five hub gene nodes, and 4466 edges. To predict transcription factors (TFs), we submitted the hub genes to the ChIP-X Enrichment Analysis 3 (ChEA3) platform11. TFs associated with the hub genes were ranked based on their mean rank score. We selected TFs with a score of 50 or less as key predicted TFs, identifying 36 TFs in total (Figure 9B; Supplementary Table S6).

## 4 Discussion

In this study, our primarily focus was on the role of ferroptosis in the development of sepsis. Bacterial infection is the main cause of sepsis (Xl et al., 2022). The occurrence of ferroptosis in intestinal epithelial cells can potentially disrupt the integrity of the intestinal barrier, facilitating the translocation of harmful intestinal bacteria and toxins into the bloodstream and extraintestinal tissues in patients with severe pancreatitis (Ma et al., 2021). During the initial stages of the immune response, there is a significant increase in iron and lipid peroxidation in macrophages. Ferroptosis inducers (RSL3, SSP, APAP) enhance the ability of macrophages to eradicate bacterial (Ma et al., 2022). Lipid peroxidation in T cells triggers ferroptosis, which can compromise the immune response against infections. The role of Gpx4 is vital for the survival of CD8^+^ T cells, as well as the proliferation of CD4^+^ and CD8^+^ T cells during infection. Gpx4 achieves this by preventing membrane lipid peroxidation and ferroptosis upon TCR activation. Ferroptosis possesses a dual role in the context of sepsis. It can facilitate bacterial invasion and sepsis induction, but it can also lead to immune cell death, compromising immune function. However, ferroptosis can also aid immune cells in eradicating pathogens. Therefore, maintaining a delicate balance between these two effects is crucial to ensure their positive contributions.

This study has identified key genes associated with ferroptosis, also known as iron death, in pediatric sepsis and has explored the potential pathogenesis of this process. This study identified a total of 74 DE-FRGs, including 60 upregulated genes and 14 downregulated genes, which were significantly expressed in peripheral blood samples of sepsis, and also identified five hub genes. However, further research is needed to confirm the extent to which these genes are involved in the pathogenesis of pediatric sepsis. Gene Ontology (GO) enrichment analysis revealed that these DE-FRGs are primarily enriched in processes such as autophagy, protein kinase activity, and ROS (Figure 3A). Interestingly, GO Molecular Function (GO-MF) enrichment also indicated a relationship between these DE-FRGs and iron ion binding (Figure 3C), which aligns with our expectations. KEGG pathway analysis showed that DE-FRGs are mainly enriched in autophagy, NLRP, and FoxO signaling pathways (Figure 3D). In the protein-protein interaction (PPI) network of DE-FRGs, five out of the 74 genes (MAPK3, MAPK8, PPARG, PTEN, and STAT3) scored higher in Cytohubba’s three algorithms.

Ferroptosis is driven by the peroxidation of specific lipids. Glutathione peroxidase 4 (GPX4) functions both as a structural protein and an antioxidant enzyme, effectively inhibiting lipid oxidation (Liu et al., 2023). In recent years, GPX4 has been recognized as a pivotal regulator of iron homeostasis. Numerous studies have demonstrated that the knockout of GPX4 can directly induce ferroptosis (Li et al., 2023; Zhang et al., 2020; Miao et al., 2022; Hangauer et al., 2017). A reduction in GPX4 activity is considered a hallmark of ferroptosis (Shen et al., 2023).

In this study, we observed that GPX4 expression was suppressed in the cardiac tissue of a CLP-induced mouse sepsis model, suggesting the occurrence of ferroptosis in the myocardial tissue. Through bioinformatics analysis, we identified five genes associated with ferroptosis—MAPK3, MAPK8, PPARG, PTEN, and STAT3—that may contribute to sepsis-related tissue damage. In the cardiac tissue of CLP mice, the protein expression level of MAPK8 was significantly reduced, while the level of PTEN was markedly increased. These findings are consistent with bioinformatics predictions, thereby further validating the reliability of our bioinformatics analysis.

Genetic association studies have demonstrated that multiple mechanisms, including autophagy, lipid metabolism, and protein kinases, are involved in the pathogenesis of sepsis (Atreya et al., 2020; Guillen-Guio et al., 2020; Shao et al., 2017). Although the pathogenesis of pediatric sepsis is not yet fully understood, abnormal autophagy appears to play a significant role (Weiss et al., 2020). Autophagy, which is crucial for maintaining cellular metabolism and organelle homeostasis, may play a role in sepsis by alleviating the condition and its associated organ damage when upregulated (Huang et al., 2023; Wu, et al., 2021). Given the anti-inflammatory effect of protein kinases observed in the experimental sepsis model of young mice, we speculate that protein kinases may improve the progression of pediatric sepsis (Inata et al., 2018). The NLRP3 inflammasome, a characteristic member of the NLR family, has been shown in numerous studies to be activated in response to reactive oxygen species during sepsis. FoxO, which regulates cellular aging mechanisms such as oxidative stress inhibition and autophagy, may also be related to pediatric sepsis and aging based on the activity level of the FoxO pathway (Bouwman and Verhaegh, 2021). The results of these GO and KEGG pathway analyses suggest that the DE-FRGs identified in this study may be involved in the onset and progression of pediatric sepsis through the aforementioned mechanisms.

In sepsis, the overproduction of reactive oxygen species (ROS) has been confirmed. Inhibiting the production of ROS can reduce the inflammatory response and improve various organ damage caused by sepsis (Li et al., 2022; Lu et al., 2022). Simultaneously, an excessive production of ROS and a decrease in the components of the antioxidant system can easily trigger ferroptosis (Park and Chung, 2019). PTEN, a well-known classic tumor suppressor gene, primarily functions as a lipid phosphatase. It dephosphorylates the substrate phosphatidylinositol (3,4,5)-trisphosphate (PIP3) into phosphatidylinositol (4,5)-bisphosphate (PIP2), acting as a negative regulator of the PI3K/Akt signaling pathway. ROS can induce the oxidation of PTEN, form intramolecular disulfide bonds, and inactivate the phosphatase function of PTEN (Nguyen Huu et al., 2021). Research has found that PPARG can reduce pyroptosis and liver damage during sepsis by inhibiting ROS levels and suppressing the TXNIP/NLRP3 signaling pathway (Li et al., 2022). Overactivation of the MAPK pathway may promote the production of ROS and induce cell death by inhibiting cysteine (Cys2) or mitochondrial CDAC2/3 12. Furthermore, MAPK is involved in iron metabolism-related inflammatory signal transduction. For instance, pretreating macrophages with ferritin light chain (FTL1) can inhibit LPS-induced ERK1/2 and JNK activation, reduce the release of NF-KB-mediated inflammatory factors, and improve the survival rate of septic mice (Zarjou et al., 2019). Therefore, the roles of MAPK3 and MAPK8 in regulating ferroptosis, sepsis inflammatory injury mechanisms, and immune responses warrant further exploration.

In this study, to further investigate the impact of immune cell infiltration in pediatric sepsis, the CIBERSORT program was used to analyze the immune infiltration process during pediatric sepsis. We observed changes in various immune cell infiltrations, which may be associated with the onset and progression of pediatric sepsis. Monocytes and macrophages play an important role in the pathophysiological process of sepsis and inflammation (Novakovic et al., 2016). The systemic inflammatory response, triggered by innate immune cells in peripheral blood during sepsis, can also influence tissue-resident immune cells, thereby contributing to organ damage in sepsis (Hoyer et al., 2019). The development of sepsis is also linked to a notable reduction in lymphocytes, characterized by a decrease in the count of CD8, CD4, B cells, and NK cells (Giza et al., 2016). Our study revealed a significant reduction in the abundance of CD4 and CD8 lymphocytes in pediatric sepsis samples. This finding may be associated with the construction of the immune microenvironment in sepsis.

This study does have certain limitations. Firstly, it relies on the GEO database, which is a secondary mining and analysis database of previously published datasets. As a result, our experimental findings may diverge from the conclusions of prior studies, likely due to the small sample size introducing bias into our data analysis. Secondly, the CIBERSORT deconvolution algorithm we used is based on a limited set of genetic data. The variability in disease-inducing factors and the plasticity of disease phenotypes could potentially lead to inaccuracies in our results. Lastly, this study only validated the occurrence of ferroptosis and the expression changes of core ferroptosis genes in the cardiac tissue of septic mice. Whether this phenomenon has clinical relevance remains to be further investigated. Despite these limitations, our study may still offer valuable insights into the potential roles of the identified immune infiltrating cells or immune-related genes in the treatment and diagnosis of sepsis, paving the way for future research into ferroptosis.

## 5 Conclusion

In conclusion, our study successfully identified MAPK3, MAPK8, PPARG, PTEN, and STAT3 as potential ferroptosis-related genes involved in the progression of sepsis. However, our understanding of the molecular mechanisms underlying ferroptosis in sepsis is still in its infancy. Further research is required to elucidate the precise molecular mechanisms involved in ferroptosis, which will provide additional evidence for the potential role of ferroptosis in the prevention and treatment of sepsis. Our study offers novel insights into the relationship between ferroptosis and sepsis, paving the way for future investigations in this area.

## Data Availability

The original contributions presented in the study are included in the article/Supplementary Material, further inquiries can be directed to the corresponding authors.

## Supplementary material

SUPPLEMENTARY FIGURE 1 Enrichment analysis of DEGs. **(A)** Top 5 GO (gene ontology) biological processes, cellular component, molecular function pathway. **(B)** Top 10 KEGG pathway.

SUPPLEMENTARY FIGURE 2 Investigation of the relationship between the hub genes and DE-MFRGs. **(A)** Heatmap displayed the expression of the DE-MFRGs. **(B)** Heatmap indicating the correlation between the hub genes and DE-MFRGs.
