# Supplementary figures and images for: Construction of a novel gene signature linked to ferroptosis in pediatric sepsis
Source: Front Cell Dev Biol. 2025 Feb 25;13:1488904. doi: 10.3389/fcell.2025.1488904 (PMC11893615; doi:10.3389/fcell.2025.1488904)

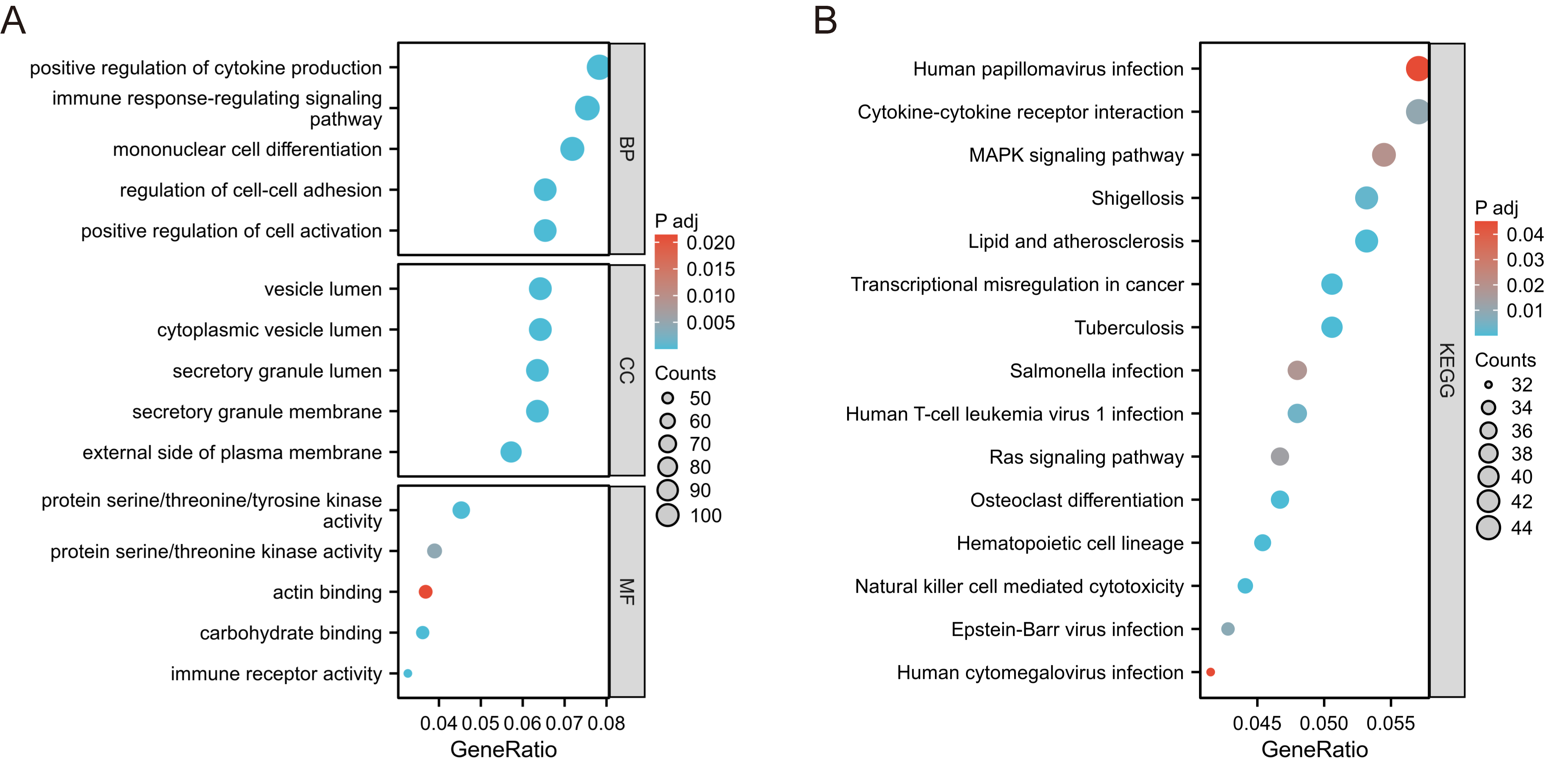

Supplement: Supplementary file 1 [file Image2.tif]

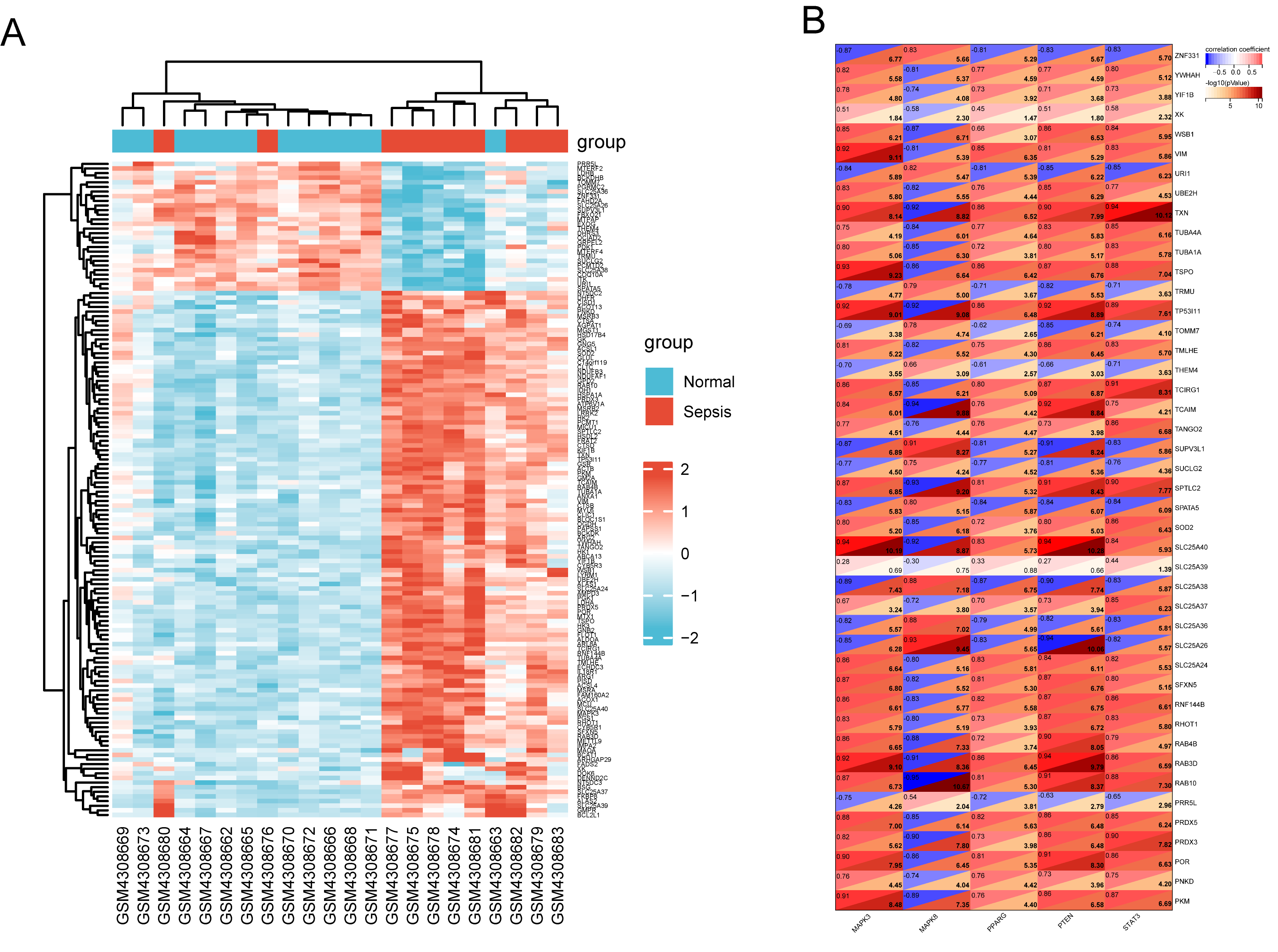

Supplement: Supplementary file 2 [file Image1.tif]
